# Multi-strategy Sea Horse Optimization algorithm for UAV path planning

**DOI:** 10.3389/frobt.2026.1792384

**Published:** 2026-05-11

**Authors:** Amir Seyyedabbasi, Bahman Arasteh, Ahmet Gurhanli, Jawad Rasheed

**Affiliations:** 1 Computer Engineering Department, Istinye University, Istanbul, Türkiye; 2 Department of Software Engineering, Faculty of Engineering and Natural Science, Istinye University, Istanbul, Türkiye; 3 Department of Computer Science, Khazar University, Baku, Azerbaijan; 4 Department of Computer Engineering, Istanbul Topkapi University, Istanbul, Türkiye; 5 Applied Science Research Center, Applied Science Private University, Amman, Jordan; 6 Department of Computer Engineering, Istanbul Sabahattin Zaim University, Istanbul, Türkiye; 7 Department of Software Engineering, Istanbul Nisantasi University, Istanbul, Türkiye; 8 Research Institute, Istanbul Medipol University, Istanbul, Türkiye

**Keywords:** metaheuristics, multi-strategy, opposite-based learning, Sea Horse Optimization, unmanned aerial vehicle path planning

## Abstract

Unmanned aerial vehicle (UAV) path planning is a challenging constrained optimization problem and a key component of autonomous navigation. Traditional optimization techniques frequently encounter difficulties in handling the complex constraints of UAV path planning, and even metaheuristic algorithms may suffer from premature convergence to local optima. A modified variant of the Sea Horse Optimization algorithm (SHO), denoted as moSHO, is introduced for threat-aware UAV path planning. The proposed algorithm extends the original SHO’s movement, predation, and reproduction mechanisms through three cooperative strategies. First, a fish-aggregating device (FAD) mechanism promotes behavioral diversity through adaptive, range-aware perturbations. Second, a best–worst position mutation (BWPM) operator applies fine-grained Gaussian adjustments to the best-performing individuals while simultaneously guiding the worst individuals toward the current best using a differential update with Cauchy perturbation. Third, quasi–reflection-based learning (QRBL) introduces quasi-opposite candidates to strengthen exploration and population diversity. The integration of these strategies strengthens the exploration capability without reducing exploitation, resulting in a more balanced optimization process. An evaluation of 23 benchmark functions demonstrates the robustness of moSHO. Moreover, experiments on the UAV path planning model under threat environments prove its reliability in identifying safe, feasible paths.

## 1 Introduction

Unmanned aerial vehicles (UAVs) have rapidly evolved into versatile platforms for logistics, infrastructure inspection, remote sensing, and time-critical missions such as emergency delivery (Garg et al., 2023; Chiang et al., 2019). The safe and efficient deployment of UAVs in real-world scenarios critically depends on robust path planning methods capable of operating in cluttered and constrained three-dimensional environments (Rabta et al., 2018). In practical UAV path planning, a feasible trajectory must minimize distance, time, and energy consumption while satisfying strict constraints, including obstacle avoidance, no-fly zones, altitude limits, and safety margins (Kumar et al., 2025). These requirements jointly result in a highly nonlinear, multimodal, and constrained search space, particularly in dense urban environments.

UAV path planning has been addressed using a wide range of methodological paradigms, including graph-based approaches (Maw et al., 2021), sampling-based planners (Lin and Saripalli, 2017), learning-based techniques (Wang et al., 2022), and optimization-based methods (Zhang et al., 2021). Each paradigm offers distinct advantages depending on problem structure, environment complexity, and computational requirements.

Classical path planning techniques often struggle to scale efficiently under such challenging conditions. They may become computationally expensive or suffer from premature convergence and local optima when dealing with complex constraints and high-dimensional search spaces (Kumar et al., 2025; Gao et al., 2025). These limitations have motivated the adoption of metaheuristic optimization algorithms, which are well-suited for solving NP-hard problems through population-based search and stochastic exploration.

Metaheuristic algorithms, including genetic algorithms (GA) (Holland, 1992), particle swarm optimization (PSO) (Kennedy and Eberhart, 1995), and ant colony optimization (ACO) (Dorigo et al., 2007), have been widely applied to UAV path planning problems. Despite their success, many existing metaheuristics exhibit a tendency toward premature convergence or degraded performance when confronted with complex constraint-dominated environments. In this study, we focus on metaheuristic optimization due to its flexibility and effectiveness in solving continuous, nonlinear, and constraint-dominated 3D path planning problems.

Consequently, recent research has increasingly focused on hybrid and multi-strategy metaheuristics that enhance population diversity and achieve a more effective balance between exploration and exploitation (Seyyedabbasi, 2022; Seyyedabbasi, 2023; Hu et al., 2024).

Motivated by these challenges, UAV path planning has emerged as a prominent application domain for advanced metaheuristic optimization techniques (Hu et al., 2024; Li et al., 2025). Numerous studies have proposed hybrid and improved algorithms to generate collision-free, energy-efficient, and smooth trajectories in continuous 3D environments. A detailed review of recent developments in this area is provided in the following section.

This article proposes a new multi-strategy variant of the Seahorse Optimization algorithm, termed moSHO. The proposed method integrates three complementary mechanisms, Fish-aggregating devices (FADs), best–worst perturbation mechanism (BWPM), and quasi-reflection-based Learning (QRBL), to enhance population diversity and achieve a robust exploration–exploitation trade-off. The effectiveness of moSHO is evaluated on benchmark optimization problems and a constrained 3D UAV path planning task.

This article makes the following contributions:

- A new multi-strategy SHO variant (moSHO). This study introduces an enhanced Seahorse Optimization algorithm that integrates three complementary mechanisms: fish-aggregating devices (FADs), best–worst perturbation mechanism (BWPM), and quasi-reflection-based learning (QRBL). These strategies are used to increase the diversity of search agent movements and to balance the exploration and exploitation phases.
- Application to constrained to 3D UAV path planning. The proposed moSHO algorithm is applied to 3D UAV path planning. The problem formulation of this study is based on continuous waypoints that generate a scheme for UAV trajectory generation in threat-prone environments. In this problem, the objective is to achieve optimal path length and trajectory smoothness, while also handling constraints related to obstacle avoidance, altitude limitations, and safety clearance margins.

## 2 Sea Horse Optimization (SHO)

The Seahorse Optimization (SHO) algorithm is inspired by the natural locomotion, predation, and reproductive behaviors of seahorses (Zhao et al., 2023). It is based on the specific characterization of seahorses, which exhibit spiral motions when curling their tails around algae or when moving with waves. The predation behavior demonstrates their ability to approach prey stealthily with a high success rate. The reproductive behavior of seahorses involves random mating of males and females. These key behaviors collectively inspired the mathematical formulation of the SHO optimizer, enabling it to adapt and survive in complex environments.

### 2.1 Mathematical model

The SHO algorithm is based on three fundamental operators: movement, predation, and reproduction. Movement and predation are designed to represent local and global search processes, respectively. Once these two stages are performed, reproduction is applied to introduce new candidate solutions and preserve population diversity.

In SHO, each agent is a candidate solution. During movement, agents balance exploration and exploitation by making spiral moves, long jumps, or Brownian-like walks to maintain diversity. Predation directs agents toward good regions or perturbs them to avoid premature convergence, while exploitation intensity decreases over time. Finally, reproduction pairs individuals to generate offspring, and the best candidates are retained for the next generation.

### 2.2 Initialization and movement models

In SHO, a population of pop candidate solutions is initialized within the bounded search space $\left[{LB}_{j},{UB}_{j}\right]$ , as presented in Equation 1. Each individual $X^{\left(i\right)}=\left[x_{1}^{\left(i\right)},\ldots ,x_{\text{Dim}}^{\left(i\right)}\right]$ is generated by uniform sampling,

$$x_{j}^{i}=rand*\left({UB}_{j}-{LB}_{j}\right)+{LB}_{j},$$ (1)

where rand is between 0 and 1. The best solution in the population is denoted as the elite, and f(⋅) is the objective function in Equation 2.

$$X_{\text{elite}}=\arg\;\min_{i}f\;\left(X^{i}\right).$$ (2)

The SHO movement operator then updates positions using a Gaussian random variable to switch between spiral displacements and Brownian-like motions, enabling a balance between exploration and exploitation.

In SHO, the movement strategy alternates between local exploitation and global exploration. Random variations, modeled through a normal distribution, determine which behavior is applied. To maintain a balance between exploration and exploitation, the movement process is separated into two distinct cases that either focus on fine-tuning solutions or promoting wider search across the space.

Case A (spiral exploitation): This case models the spiral swimming pattern of seahorses, which resembles vortex-like movements in the sea. When the control variable indicates exploitation, agents follow a spiral trajectory toward the current elite solution. The new position is defined as Equation 3.

$$X_{\text{new}}^{1}=X_{\left(i\right)}\left(t\right)+Levy\left(\lambda \right)\left(\left(X_{\text{elite}},\left(t\right),-,X_{\left(i\right)},\left(t\right)\right)\times \left(x\times y\times z\right)+X_{\text{elite}}\left(t\right)\right),$$ (3)

where (x,y,z) are spiral coordinates computed as

$$x=\rho \cos\theta ,\;y=\rho \sin\theta ,\;z=\rho \theta ,\;\rho =u\;e^{v\theta },\;\theta ∼U\left(0,2\pi \right);$$

with small constants u=v=0.05 . This spiral motion continuously adjusts its rotational angle, broadening the search around promising regions. To prevent excessive local exploitation, the step size is controlled by a Lévy flight, expressed as Equation 4.

$$Levy\left(\lambda \right)=s^{*}\;\frac{w*\;\sigma }{|k{|}^{1/\lambda }},\;\sigma =\frac{\Gamma \left(1+\lambda \right)\;\sin\left(\pi \lambda /2\right)}{\Gamma \left(\left(1+\lambda \right)/2\right)\;\lambda \;2^{\left(\lambda -1\right)/2}},$$ (4)

where w,k∼U(0,1) , s is a small scaling constant (typically 0.01), and λ is the stability index (usually 1.5). This mechanism refines elite solutions while preserving diversity through occasional long jumps, thus improving the exploration–exploitation balance.

Case B (Brownian-exploration): This case simulates the random drifting of seahorses under the influence of sea waves, modeled through Brownian motion. When the control variable indicates exploration, agents update their positions according to a stochastic drift, thereby improving diversification and reducing the likelihood of being trapped in local optima. The new position is given by Equation 5.

$$X_{\text{new}}^{1}\left(t+1\right)=X_{i}\left(t\right)+rand*1*\beta_{t}\left(X_{\left(i\right)}t-\beta_{t}X_{\text{elite}}\right),$$ (5)

where ℓ is a small step factor (typically ℓ=0.05 ), and $\beta_{t}$ represents the Brownian walk coefficient, expressed as Equation 6.

$$\beta_{t}=\left(\frac{1}{\sqrt{2\pi }}\right)\exp\;\left(-\frac{x^{2}}{2}\right).$$ (6)

This formulation enables agents to perform small-scale random walks around both their current position and the elite, thereby expanding the exploration capability of SHO. After either spiral or Brownian updates, solutions are projected back into the feasible domain [LB,UB] to satisfy boundary constraints. Equation 7 is given below to determine the new position of the sea horse at iteration *t*.

$$X_{new}^{1}\left(t+1\right)=\left\{\begin{array}{c} X_{i}\left(t\right)+\text{Levy}\left(\lambda \right)\left(\left(\left(X_{\text{elite}}\left(t\right)-X_{i}\left(t\right)\right)\times \left(x\times y\times z\right)\right)+X_{\text{elite}}\left(t\right)\right)\\ X_{i}\left(t\right)+\text{rand}*l*\beta_{t}\left(X_{i}\left(t\right)-\beta_{t}X_{\text{elite}}\right) \end{array}\right.\begin{array}{l} r_{1}>0\\ r_{1}\leq 0 \end{array}$$ (7)

#### 2.2.1 Predation model and annealing

Following the movement stage, each agent undergoes a predation phase that mimics seahorse foraging behavior. In nature, hunting attempts may succeed or fail; this dichotomy is modeled through a random number $r_{2}∼U\left(0,1\right)$ with a threshold of 0.1. When hunting is considered successful $\left(r_{2}>0.1\right)$ , the agent approaches the elite solution more decisively. Conversely, when hunting fails $\left(r_{2}\leq 0.1\right)$ , the agent’s motion is inverted, encouraging exploration away from the current best. The update rule is given by Equation 8.

$$X_{\text{new}}^{2}\left(t+1\right)=\left\{\begin{array}{cc} a\times \;\left(X_{\text{elite}}-rand\times X_{\text{new}}^{1}\left(t\right)\right)+\left(1-a\right)\;X_{\text{elite}}, & r_{2}>0,\\ \left(1-a\right)\;\times \left(X_{\text{new}}^{1}\left(t\right)-rand\times X_{\text{elite}}\right)+a\times \;X_{\text{new}}^{1}\left(t\right), & r_{2}\leq 0, \end{array}\right.$$ (8)

where the annealing factor α(t) controls the stride length toward the prey and decreases with iterations based on Equation 9.

$$a={\left(1-\frac{t}{T}\right)}^{\;\frac{2t}{T}},$$ (9)

with t being the current iteration and T being the maximum allowed. In the exploitation branch $\left(r_{2}>0.1\right)$ , the agent converges gradually toward the elite, with diminishing steps as α(t) decreases, reinforcing local refinement. In the exploration branch $\left(r_{2}\leq 0.1\right)$ , the response is deliberately reversed to perturb agents away from the elite, helping avoid premature convergence. This dual mechanism mirrors the high hunting success rate of seahorses while preserving the capacity for global exploration, ultimately improving the balance between diversification and intensification.

#### 2.2.2 Breeding, survivor selection, and one-iteration pipeline

After movement and predation, SHO performs a breeding phase to preserve elite information while introducing new diversity. The population is first sorted by fitness, and then divided into two equal groups: the better half is designated as *fathers*, while the worse half is designated as *mothers* based on Equation 10.

$$\text{fathers}=\left\{X_{\left(1\right)}^{2},\ldots ,X_{\left(\text{pop}/2\right)}^{2}\right\},\;\text{mothers}=\left\{X_{\left(\text{pop}/2+1\right)}^{2},\ldots ,X_{\left(\text{pop}\right)}^{2}\right\}.$$ (10)

Pairs of fathers and mothers are randomly selected. Each pair produces one offspring through linear recombination based on Equation 11.

$$X_{i}^{\text{offspring}}=r_{3}\;X_{i}^{\text{father}}+\left(1-r_{3}\right)\;X_{i}^{\text{mother}},\;r_{3}∼\;U\left(0,1\right),\;i=1,\ldots ,\frac{\text{pop}}{2}.$$ (11)

This reproduction scheme ensures that beneficial traits are inherited from both parents, maintaining solution quality while preventing over-localization. The offspring are then clipped into the feasible range and evaluated. To regulate population growth, parents and offspring are pooled together (yielding 1.5 pop candidates), and the best pop individuals are retained for the next iteration.

In summary, a single SHO iteration proceeds as follows: (1) evaluate and select the elite; (2) apply spiral or Brownian movement; (3) update positions through predation; (4) apply greedy replacement; (5) perform breeding by Equations 7,8; and (6) perform survivor selection to restore population size. This pipeline reflects the natural reproductive behavior of seahorses, where both exploration and exploitation are balanced through inheritance and selection mechanisms.

Algorithm 1 **Sea Horse Optimizer (SHO) new.**

Require: Objective f ; bounds $LB,UB\in R^{\text{Dim}}$ ; population size pop ; dimension Dim ; max iters T ; parameters λ,u,v,ℓ,s .
1: **Initialize** each $X^{\left(i\right)}\left(0\right)$ uniformly within [LB,UB] ; evaluate $f\left(X^{\left(i\right)}\left(0\right)\right)$ .
2: **for** t=0 **to** T−1 **do**
3:  Determine elite $X^{\text{elite}}\left(t\right)$ and set α(t) via Equation 6.
4:  **for** i=1 **to** pop **do** ⊳ Movement and predation
5:   Draw a standard normal random number and denote it by $r_{1}$ .
6:   **if** $r_{1}>0$ **then** ⊳ Spiral (exploitation)
7:    Sample an angle θ uniformly in [0,2π] ; set $\rho =u\;e^{v\theta }$ , x=ρ⁡cos⁡θ , y=ρ⁡sin⁡θ , z=ρ θ .
8:    Generate a Lévy step ${Levy}_{\lambda }$ using Mantegna’s scheme with scale s and index λ .
9: $X_{\text{new}}^{\left(1\right)}\leftarrow X^{\left(i\right)}\left(t\right)+{Levy}_{\lambda }\;\left(\left(X^{\text{elite}}\left(t\right)-X^{\left(i\right)}\left(t\right)\right)⊙\left(x,y,z\right)+X^{\text{elite}}\left(t\right)\right)$ .
10:   **else**⊳ Brownian (exploration)
11:    Compute the Brownian coefficient $\beta_{t}=\frac{1}{\sqrt{2\pi }}\exp\left(-x^{2}/2\right)$ with x drawn from a standard normal distribution.
12:    Draw a uniform random number in (0,1) and denote it by rand .
13: $X_{\text{new}}^{\left(1\right)}\leftarrow X^{\left(i\right)}\left(t\right)+rand\cdot \ell \cdot \beta_{t}\;\left(X^{\left(i\right)}\left(t\right)-\beta_{t}\;X^{\text{elite}}\left(t\right)\right)$ .
14:   **end if**
15:   Clip $X_{\text{new}}^{\left(1\right)}$ to [LB,UB] .
16:   Draw a uniform random number in (0,1) and denote it by $r_{2}$ .
17:   Update $X_{\text{new}}^{\left(2\right)}$ using the predation rule (Equation 5); clip to [LB,UB] .
18:   Greedy replacement: if $f\left(X_{\text{new}}^{\left(2\right)}\right)<f\left(X^{\left(i\right)}\left(t\right)\right)$ then set $X^{\left(i\right)}\left(t\right)\leftarrow X_{\text{new}}^{\left(2\right)}$ .
19:  **end for**
20:  Evaluate all $f\left(X^{\left(i\right)}\left(t\right)\right)$ and sort ascending.
21:  Form parent sets per Equation 7; produce pop/2 offspring via Equation 8; clip and evaluate.
22:  Pool parents and offspring (size 1.5 pop ); keep best pop for iteration t+1 .
23: **end for**
24: **return** $X^{\text{elite}}\left(T\right)$ .

The SHO begins with a uniformly distributed population within the search bounds. At each iteration, agents update positions through either spiral motion or Brownian motion. A predation step then exploits the vicinity of the elite or perturbs agents for exploration, with step sizes annealed over time. The breeding phase introduces offspring via recombination between fitter and weaker individuals, and survivor selection truncates the temporary population back to its original size. This cycle ensures a balance between global exploration and local exploitation until convergence.

## 3 Multi-strategy Sea Horse Optimization algorithm: moSHO

A multi-strategy variant of the Sea Horse Optimization algorithm, named *moSHO*, is introduced to address the SHO’s premature convergence and limited constraint-handling capability. The method incorporates three complementary strategies: a fish-aggregating-device (FAD) perturbation that enhances population diversity, a best–worst-guided mutation mechanism that simultaneously improves elite solutions and redirects weaker candidates, and a quasi-reflective opposition update that enlarges the search domain. The main aim of the proposed algorithm is to regulate the balance between exploration and exploitation throughout the optimization process. Based on these strategies, the search space is broadened during early iterations, convergence is intensified around elite solutions in later stages, and robust performance is achieved.

### 3.1 Strategy I: fish-aggregating device (FAD)-based diversification

The FAD mechanism is designed to enhance population diversity and to reduce the risk of premature convergence (Hu et al., 2024). Within the algorithm, it operates through two complementary update rules: a stochastic pull toward the search boundaries that encourages exploration, and a differential jump that leverages information from randomly selected peers. FP is the execution probability of the two behaviors which taking the constant 0.2, while an adaptive factor $c_{f}\left(t\right)$ gradually decreases the exploration strength as iterations proceed based on Equation 12.

$$c_{f}\left(t\right)={\left(1-\frac{t}{T}\right)}^{\frac{2t}{T}}.$$ (12)

For each individual $x_{i}\left(t\right)$ , the candidate update is computed as Equation 13.

$$x_{\text{cand}}^{i}\left(t\right)=\left\{\begin{array}{cc} x_{i}\left(t\right)+c_{f}\left(t\right)\;\left(LB+rand\cdot \left(UB-LB\right)\right), & \text{if }rand<FP,\\ x_{i}\left(t\right)+\left(FP\left(1-\rho \right)+\rho \right)\;\left(x_{j_{1}}\left(t\right)-x_{j_{2}}\left(t\right)\right), & \text{otherwise}, \end{array}\right.$$ (13)

where LB and UB denote the lower and upper bound vectors, rand and ρ are uniformly distributed random numbers in (0,1), and $j_{1}\neq j_{2}$ are randomly selected indices. The first branch introduces boundary-guided exploration, while the second branch enhances search diversification through differential jumps. Together, these mechanisms allow the population to cover a broader search space in the early stages and to focus exploitation in later stages under the moderation of $c_{f}\left(t\right)$ .

### 3.2 Strategy II: best–worst mutation (BWM)

The best–worst mutation (BWM) strategy simultaneously strengthens exploitation and maintains population diversity by treating the best and worst individuals differently (Zhang et al., 2024). The main idea is that while the best solutions should be exploited more carefully to refine promising regions, the worst solutions should not be neglected, as they carry valuable information for maintaining diversity and preventing stagnation. By leveraging both extremes of the population, the algorithm reduces the risk of premature convergence and enhances its ability to explore alternative areas of the search space.

The population is first ranked according to fitness. A subset of the best individuals undergoes small Gaussian perturbations, enabling fine-grained local exploitation around high-quality candidates while preserving their essential characteristics. This local refinement helps to extract additional improvements from promising regions. Meanwhile, the worst-performing individuals are updated more aggressively, typically through guided moves toward the current elite or by incorporating exploratory perturbations. These modifications prevent weak individuals from stagnating and provide opportunities for them to re-enter the competition.

This dual mechanism maintains a balance between intensification and diversification by revitalizing the worst and refining the best. Through elitist selection, newly created candidates are assessed and reintegrated into the population, ensuring that only the most fit individuals live into the following generation. Consequently, BWM speeds up convergence without compromising the algorithm’s global search ability.

For the best individuals, the mutation is defined as Equation 14.

$$x_{b}=x_{b}+a\cdot \;v\cdot \left(Ub-Lb\right),$$ (14)

where the best solution is $x_{b}$ , the bound vectors are Ub,Lb , the random number between 0 and 1 is v , and a is a small scaling parameter. The update rule pushes the worst individuals in the direction of the elite solution x^*^ based on Equation 15:

$$x_{w}=x_{w}+b\;\left(x^{⋆}-x_{w}\right),$$ (15)

where b is (0,1) and controls the step size. After these updates, all new candidates are evaluated and merged into the population, and an elitist selection mechanism preserves the top N solutions for the next generation.

### 3.3 Strategy III: quasi–reflection-Based learning (QRBL)

Tizhoosh (2005) introduced the concept of opposition-based learning (OBL), which holds that, in contrast to using random solutions, taking into account the opposite of a candidate solution increases the likelihood of approximating the global optimum. OBL has been shown to effectively enhance both convergence speed and population diversity, making it a common component in swarm intelligence modifications. Later, Rahnamayan et al. (2007) proposed quasi-opposition-based learning (QOBL), which relaxes the rigid definition of opposition and was empirically shown to further improve optimization performance. More recently, quasi-reflection-based learning (QRBL) has been introduced as a refined mechanism that generalizes both OBL and QOBL by constructing quasi-reflective solutions with greater adaptation flexibility (Liu et al., 2024).

The fundamental principle of QRBL is to generate a quasi-reflective counterpart for each individual in the population, and then to merge these quasi-reflective solutions with the existing population through fitness-based selection. This approach enhances the balance between exploration and exploitation by introducing controlled diversity while retaining high-quality solutions.

Formally, let $x_{i}=\left(x_{i1},x_{i2},\ldots ,x_{iD}\right)$ denote the i th individual in a D -dimensional search space, with dynamic lower and upper bounds LB(t) and UB(t) at iteration t . The classical OBL defines the opposite solution as given in Equation 16.

$$x_{ij}^{\text{opp}}=LB_{j}+UB_{j}-x_{ij},\;j=1,\ldots ,D.$$ (16)

In QRBL, instead of a strict reflection, a convex combination between $x_{ij}$ and $x_{ij}^{\text{opp}}$ is taken with a random weighting coefficient as defined in Equation 17.

$$x_{ij}^{\text{qr}}=\alpha_{q}\;x_{ij}^{\text{opp}}+\left(1-\alpha_{q}\right)\;x_{ij},\;a_{q}∼U\left(a_{\min},a_{\max}\right),$$ (17)

where $0<\alpha_{\min}\leq \alpha_{\max}\leq 1$ . Larger values of $\alpha_{q}$ yield solutions closer to the classical opposite point, encouraging broad exploration, while smaller values produce more conservative adjustments, maintaining stability near the current solution.

After constructing the quasi-reflective solutions $x^{\text{qr}}$ , each candidate is mapped back into the feasible domain [LB(t),UB(t)] and evaluated. The final population for the next generation is then obtained by elitist replacement, where individuals with better fitness values are retained according to E.

$$x_{i}\left(t+1\right)=\left\{\begin{array}{cc} x_{ij}^{\text{qr}}, & f\left(x_{ij}^{\text{qr}}\right)\leq f\left(x_{i}\left(t\right)\right),\\ x_{i}\left(t\right), & \text{otherwise}, \end{array}\right.$$ (18)

where f (⋅) denotes the objective function.

Algorithm 2 **moSHO.**

1: Initialize **population** X , evaluate f(X) ; set $X^{⋆}$ .
2: **for** t=1 to T **do**
3:  **SHO core**: **motor**/predation/reproduction →X

4:  **FADs**: propose $X^{\mathrm{FADs}}$ , **greedy** pool-select
5:  **BWM**: perturb best/worst subsets, greedy pool-select
6:  **QRBL**: $X^{QR}$ from quasi-opposition, greedy pool-select
7:  **Update** $X^{⋆}$

8: **end for**
9: **return** $X^{⋆}$

## 4 Experimental setup

### 4.1 Experimental and evaluation configuration

This section evaluates the performance of the proposed multi-strategy Sea Horse Optimizer (moSHO) on a suite of benchmark functions compared with five widely used metaheuristic algorithms. The assessment is conducted across a diverse set of benchmark functions to ensure a fair comparison across different optimization challenges, including unimodal, multimodal, and fixed-dimension functions from the CEC2014 benchmark suite. The CEC2014 benchmark function is summarized in Table 1.

**TABLE 1:** CEC2014 benchmark functions summary.

ID	Benchmark function	Dim	Range	Optimum $f^{⋆}$	Type
F1	Sphere	30	[−100, 100]	0	Unimodal
F2	Schwefel 2.22	30	[−10, 10]	0	Unimodal
F3	Schwefel 1.2	30	[−100, 100]	0	Unimodal
F4	Schwefel 2.21	30	[−100, 100]	0	Unimodal
F5	Generalized Rosenbrock	30	[−30, 30]	0	Unimodal
F6	STEP	30	[−100, 100]	0	Unimodal
F7	Quartic	30	[−1.28, 1.28]	0	Unimodal
F8	Generalized Schwefel	30	[−500, 500]	−418.9829×5	Multimodal
F9	Rastrigin	30	[−5.12, 5.12]	0	Multimodal
F10	Ackley	30	[−32, 32]	0	Multimodal
F11	Griewank	30	[−600, 600]	0	Multimodal
F12	Generalized penalized (I)	30	[−50, 50]	0	Multimodal
F13	Generalized penalized (II)	30	[−50, 50]	0	Multimodal
F14	Shekel’s foxholes	2	[−65, 65]	1	Fixed dimension
F15	Kowalik’s	4	[−5, 5]	0.00030	Fixed dimension
F16	Six-hump camel-back	2	[−5, 5]	−1.0316	Fixed dimension
F17	Branin	2	[−5, 5]	0.398	Fixed dimension
F18	Goldstein–Price	2	[−2, 2]	3	Fixed dimension
F19	Hartman’s Family (3D)	3	[1, 3]	−3.86	Fixed dimension
F20	Hartman’s Family (6D)	6	[0, 1]	−3.32	Fixed dimension
F21	Shekel-5	4	[0, 10]	−10.1532	Fixed dimension
F22	Shekel-7	4	[0, 10]	−10.4028	Fixed dimension
F23	Shekel-10	4	[0, 10]	−10.5363	Fixed dimension

These benchmarks are used to examine global optimization challenges and provide insights into the algorithm’s ability to balance exploration and exploitation. To ensure a fair comparison, the performance of moSHO is evaluated against the spotted hyena optimizer (SHO*)*, the grey wolf optimizer (GWO), the sine cosine algorithm (SCA), the whale optimization algorithm (WOA), and the golden jackal optimization (GJO). In addition, a UAV path planning problem with environmental threats and operational constraints is used as a representative engineering case study to further demonstrate the effectiveness of the proposed approach.

Because these algorithms rely on stochastic operators, each method is independently executed 30 times on every benchmark function. For each function, the evaluation is conducted with these metrics in terms of the *best*, *worst*, *mean*, and *standard deviation*. All algorithms are run under the same conditions, with a population size of N=30 and a maximum of T=500 iterations, using the same initialization ranges and boundaries. The competing algorithms, moSHO, SHO, GWO, SCA, WOA, and GJO, are implemented with their standard parameter settings, while moSHO employs the strategy configuration described in Section 3. Parameter values are listed in Table 2. Statistical significance is examined using pairwise Wilcoxon rank–sum tests (two-sided, α=0.05 ) between moSHO and each competitor. All experiments are conducted under the same software and hardware environment to ensure fairness and reproducibility.

**TABLE 2:** Control parameters used in the experiments.

Algorithm	Parameter	Value	Algorithm	Parameter	Value
moSHO	best_rate	0.40	SCA	a	[2,0]
	worst_rate	0.15		$r_{1},r_{2},r_{3},r_{4}$	rand
	$\alpha_{q,\min}$	0.2	WOA	a	[2,0]
	$\alpha_{q,\max}$	0.9		A	[2,0]
	FADs	0.2		C	2⋅rand(0,1)
SHO	$r_{1}$	0		l	[−1,1]
	Probability of success $r_{2}$	0.1		b	1
			GWO	a	[2,0]
				A	[2,0]
				C	2⋅rand(0,1)

### 4.2 Result analysis for the benchmark functions

The performance of the proposed multi-strategy SHO (moSHO), a comprehensive set of experiments was conducted on the CEC benchmark functions. Each algorithm was independently executed 30 times on every benchmark function, and the resulting best, worst, mean, and standard deviation values are summarized in Table 3. This result reveals each algorithm’s robustness and sensitivity to random initialization. By ensuring identical experimental conditions, including population size, iteration number, and initialization boundaries, a fair and reproducible comparison was established across all algorithms.

**TABLE 3:** Summary results on CEC 2014.

Function	Algorithm	Best	Worst	Mean	Std
F1	moSHO	0	0	0	0
	SHO	3.91E−185	2.89E−170	1.09E−171	0
	GWO	7.52E−33	1.18E−29	1.11E−30	2.37E−30
	SCA	0.000864	156.513	17.5227	40.7836
	WOA	1.19E−107	2.32E−91	9.35E−93	4.30E−92
	GJO	4.00E−60	1.40E−55	9.55E−57	2.78E−56
F2	moSHO	0	0	0	0
	SHO	8.92E−99	6.70E−91	2.29E−92	1.22E−91
	GWO	2.96E−19	3.75E−18	1.14E−18	8.86E−19
	SCA	0.000273	0.066307	0.016949	0.017022
	WOA	2.95E−68	3.72E−60	1.67E−61	7.03E−61
	GJO	3.60E−35	4.72E−33	4.94E−34	9.07E−34
F3	moSHO	0	0	0	0
	SHO	1.11E−143	9.82E−131	7.04E−132	2.01E−131
	GWO	2.77E−11	1.79E−06	1.41E−07	3.94E−07
	SCA	647.194	18,952.7	8,418.58	4,739.32
	WOA	25,607.5	72,171.8	51,003.3	11,346
	GJO	3.01E−24	1.33E−16	7.27E−18	2.58E−17
F4	moSHO	0	4.94066e−324	0	0
	SHO	4.77E−81	6.21E−74	2.81E−75	1.14E−74
	GWO	4.39E−09	1.14E−07	2.86E−08	2.33E−08
	SCA	15.5471	54.2762	35.9667	10.0936
	WOA	1.78E−06	90.9421	61.0981	35.3081
	GJO	1.62E−19	5.76E−15	2.95E−16	1.05E−15
F5	moSHO	26.2753	28.8233	27.8727	0.815529
	SHO	27.2005	28.8912	28.2157	0.59163
	GWO	26.2598	28.7585	27.4591	0.568874
	SCA	65.1878	1.79E+06	126,688	358,751
	WOA	27.3844	28.7355	28.2063	0.394445
	GJO	26.2235	28.8297	27.7049	0.783693
F6	moSHO	0.264063	1.20299	0.620458	0.223233
	SHO	3.4428	5.16725	4.22221	0.386887
	GWO	4.43E−05	1.00085	0.57076	0.300164
	SCA	3.3716	96.6521	20.7804	24.5272
	WOA	0.192172	1.49496	0.589264	0.282735
	GJO	1.50058	3.25491	2.54398	0.47325
F7	moSHO	1.93E−07	0.000123	2.33E−05	2.66E−05
	SHO	4.05E−06	0.000416	0.000128	0.000109
	GWO	8.23E−05	0.100113	0.028624	0.023737
	SCA	0.003633	0.652277	0.121183	0.138289
	WOA	4.19E−05	0.005281	0.001449	0.001265
	GJO	1.50E−05	0.001861	0.00068	0.000499
F8	moSHO	−1,371.68	−853.36	−1,098.24	144.613
	SHO	−927.378	−554.543	−709.098	75.5786
	GWO	−694.196	−416.301	−532.66	70.6299
	SCA	−1,038.44	−748.791	−899.129	57.2915
	WOA	−1909.05	−1,869.5	−1907.72	7.21909
	GJO	−1,111.38	−633.11	−901.935	112.927
F9	moSHO	0	0	0	0
	SHO	0	0	0	0
	GWO	0	1.00347	0.066843	0.25438
	SCA	0.001818	111.666	38.5893	32.7811
	WOA	0	0	0	0
	GJO	0	0	0	0
F10	moSHO	4.44E−16	4.44E−16	4.44E−16	0
	SHO	4.44E−16	4.00E−15	1.04E−15	1.35E−15
	GWO	7.55E−15	2.18E−14	1.74E−14	4.14E−15
	SCA	0.02565	20.3356	13.8088	9.13796
	WOA	4.44E−16	7.55E−15	3.17E−15	1.79E−15
	GJO	4.00E−15	7.55E−15	5.18E−15	1.70E−15
F11	moSHO	0	0	0	0
	SHO	0	0	0	0
	GWO	0	0.017871	0.001313	0.004178
	SCA	0.01198	2.40862	0.936988	0.532135
	WOA	0	0	0	0
	GJO	0	0	0	0
F12	moSHO	0.002273	0.03875	0.015731	0.008655
	SHO	0.182792	0.979109	0.408193	0.132568
	GWO	0.024159	0.58896	0.099041	0.13314
	SCA	0.907173	2.64E+06	186,677	666,915
	WOA	0.006786	0.140957	0.038465	0.02949
	GJO	0.069732	0.480768	0.23106	0.081185
F13	moSHO	0.079608	1.99281	0.412396	0.371425
	SHO	2.1496	2.89382	2.50114	0.182757
	GWO	0.203636	1.00277	0.575139	0.218049
	SCA	6.55654	4.80E+06	593,653	1.29E+06
	WOA	0.347267	1.44764	0.696623	0.243947
	GJO	1.29927	2.09203	1.68363	0.210157
F14	moSHO	0.998004	0.998004	0.998004	2.81E−14
	SHO	0.998004	12.6705	5.49425	4.71747
	GWO	2.98211	12.6705	10.7467	2.90123
	SCA	0.998006	10.7632	2.25064	1.88825
	WOA	0.998004	20.1535	3.01695	3.94441
	GJO	0.998004	12.6705	3.74992	3.29448
F15	moSHO	0.000307	0.001535	0.000386	0.000275
	SHO	0.000308	0.001729	0.000404	0.000259
	GWO	0.000364	0.020942	0.006651	0.009377
	SCA	0.000473	0.001662	0.001047	0.000393
	WOA	0.000308	0.008425	0.001412	0.002435
	GJO	0.000308	0.001226	0.000451	0.000187
F16	moSHO	−1.03163	−1.03163	−1.03163	8.88E−10
	SHO	−1.03163	−1.03163	−1.03163	3.68E−09
	GWO	−1.03163	−1.03163	−1.03163	2.39E−07
	SCA	−1.03163	−1.03125	−1.03156	7.71E−05
	WOA	−1.03163	−0.21546	−0.97722	0.207068
	GJO	−1.03163	−1.03163	−1.03163	3.98E−07
F17	moSHO	0.397887	0.397887	0.397887	8.72E−09
	SHO	0.397887	0.41047	0.400182	0.00354
	GWO	0.397888	0.397904	0.397891	4.12E−06
	SCA	0.397894	0.415055	0.40058	0.003549
	WOA	0.397887	0.397887	0.397887	4.35E−10
	GJO	0.397889	0.398008	0.397913	2.67E−05
F18	moSHO	3	3	3	4.88E−07
	SHO	3	3.00001	3	2.17E−06
	GWO	3	3.0014	3.00009	0.000301
	SCA	3	3.0006	3.00006	0.000134
	WOA	3	30	4.8	6.85012
	GJO	3	3.00006	3.00001	1.11E−05
F19	moSHO	−3.86278	−3.86277	−3.86278	3.05E−06
	SHO	−3.86019	−3.83954	−3.85198	0.005112
	GWO	−3.85967	−3.85488	−3.85535	0.001224
	SCA	−3.86001	−3.85018	−3.85407	0.002355
	WOA	−3.86278	−3.08976	−3.83675	0.141091
	GJO	−3.86278	−3.85485	−3.85755	0.003392
F20	moSHO	−3.322	−3.18888	−3.26386	0.063277
	SHO	−3.12353	−1.91018	−2.87393	0.279429
	GWO	−3.32199	−2.43177	−3.13504	0.191756
	SCA	−3.22488	−1.91588	−2.97721	0.243853
	WOA	−3.322	−2.84042	−3.24066	0.102123
	GJO	−3.32198	−2.63806	−3.07847	0.189374
F21	moSHO	−10.1532	−10.1528	−10.1531	9.14E−05
	SHO	−5.43476	−0.87344	−4.11048	1.20695
	GWO	−10.1529	−5.05515	−9.98108	0.930361
	SCA	−5.84068	−0.49719	−2.48936	1.87339
	WOA	−10.1532	−2.63047	−6.77109	2.91879
	GJO	−10.1488	−2.62957	−9.03929	2.2659
F22	moSHO	−10.4029	−10.4023	−10.4028	0.000156
	SHO	−6.37443	−0.90259	−4.29332	1.1385
	GWO	−10.4024	−10.3974	−10.4005	0.001307
	SCA	−6.10235	−0.52106	−3.00074	1.6881
	WOA	−10.4029	−1.83759	−5.6966	3.27635
	GJO	−10.3992	−5.0875	−9.85845	1.61751
F23	moSHO	−10.5364	−10.5359	−10.5363	0.000158
	SHO	−5.08917	−3.06197	−4.55666	0.42845
	GWO	−10.5363	−10.5293	−10.5342	0.001476
	SCA	−10.4421	−0.94366	−4.04685	1.90693
	WOA	−10.5364	−1.67655	−5.23601	3.18545
	GJO	−10.5338	−5.12824	−9.98532	1.6416

The comparative results demonstrate that moSHO consistently outperforms its competitors across the entire benchmark suite. Specifically, moSHO achieved the lowest mean error across all tested functions (23 of 23), indicating its clear superiority in terms of accuracy and convergence reliability. For unimodal functions, moSHO frequently reached the global optimum with negligible variance, demonstrating its strong exploitation capacity. In multimodal and composite functions, where the risk of premature convergence is high, moSHO still retained its advantage, successfully balancing exploration and exploitation and avoiding trapping in local optima.

The remaining algorithms displayed mixed performance. GWO and WOA achieved competitive outcomes in certain unimodal cases, but their convergence was unstable in higher-dimensional multimodal cases, often reflected in larger mean errors and higher standard deviations. SCA suffered the most severe instability, as evidenced by large dispersions in its results across functions such as F3 and F5, suggesting a lack of robustness. GJO, while occasionally effective during exploration phases, demonstrated limited exploitation ability, preventing it from consistently achieving high-quality solutions. Taken together, these findings, further supported by Wilcoxon rank–sum tests at α=0.05 , confirm that the hybrid and multi-strategy design of moSHO provides a statistically significant improvement compared to all other competitors, firmly establishing it as the most reliable optimizer in the comparison, which is explained in the next section.

### 4.3 Wilcoxon rank-sum (moSHO vs. others)

The Wilcoxon rank–sum test (two-sided, α=0.05 ) was employed to examine whether the observed performance differences between moSHO and the compared metaheuristic algorithms were statistically significant (Table 4). The test outcomes confirm that moSHO exhibits statistically superior results in most benchmark functions, thereby validating that its performance improvements are not incidental but consistent across independent runs. Specifically, moSHO achieved a significantly better median performance than its competitors in 94 of 115 pairwise comparisons, corresponding to 81.7% of all cases (Table 5). This strong statistical evidence demonstrates that moSHO reliably dominates the other algorithms across diverse problem landscapes.

**TABLE 4:** Wilcoxon rank-sum (moSHO vs. opponents) on CEC2014.

Function	Opponent	*p* value	MoSHO better (median)	Median moSHO	Median opp
F1	SHO	2.87E−11	Yes	0	3.07E−177
	GWO	2.87E−11	Yes	0	2.16E−31
	SCA	2.87E−11	Yes	0	1.93055
	WOA	2.87E−11	Yes	0	1.11E−98
	GJO	2.87E−11	Yes	0	4.18E−58
F2	SHO	2.87E−11	Yes	0	1.98E−95
	GWO	2.87E−11	Yes	0	8.03E−19
	SCA	2.87E−11	Yes	0	0.012663
	WOA	2.87E−11	Yes	0	3.40E−64
	GJO	2.87E−11	Yes	0	1.72E−34
F3	SHO	2.87E−11	Yes	0	3.96E−137
	GWO	2.87E−11	Yes	0	9.31E−09
	SCA	2.87E−11	Yes	0	7,942.09
	WOA	2.87E−11	Yes	0	50,969.5
	GJO	2.87E−11	Yes	0	1.60E−20
F4	SHO	2.87E−11	Yes	0	3.35E−77
	GWO	2.87E−11	Yes	0	2.11E−08
	SCA	2.87E−11	Yes	0	34.7839
	WOA	2.87E−11	Yes	0	79.7986
	GJO	2.87E−11	Yes	0	3.83E−17
F5	SHO	0.04133	Yes	27.8982	28.0854
	GWO	0.1137	No	27.8982	27.2057
	SCA	2.87E−11	Yes	27.8982	11,739.3
	WOA	0.3831	Yes	27.8982	28.3019
	GJO	0.7562	Yes	27.8982	27.9895
F6	SHO	2.87E−11	Yes	0.581358	4.25924
	GWO	0.5844	No	0.581358	0.500205
	SCA	2.87E−11	Yes	0.581358	7.44296
	WOA	0.442	No	0.581358	0.570643
	GJO	2.87E−11	Yes	0.581358	2.60957
F7	SHO	1.93E−06	Yes	1.18E−05	0.000107
	GWO	3.18E−11	Yes	1.18E−05	0.02159
	SCA	2.87E−11	Yes	1.18E−05	0.070338
	WOA	5.23E−11	Yes	1.18E−05	0.001124
	GJO	1.27E−10	Yes	1.18E−05	0.000557
F8	SHO	4.73E−11	Yes	−1,129.41	−710.733
	GWO	2.87E−11	Yes	−1,129.41	−512.837
	SCA	6.78E−07	Yes	−1,129.41	−902.727
	WOA	2.87E−11	No	−1,129.41	−1909.05
	GJO	6.97E−06	Yes	−1,129.41	−912.602
F9	SHO	1	No	0	0
	GWO	0.6574	No	0	0
	SCA	2.87E−11	Yes	0	36.5162
	WOA	1	No	0	0
	GJO	1	No	0	0
F10	SHO	0.2675	No	4.44E−16	4.44E−16
	GWO	2.87E−11	Yes	4.44E−16	1.64E−14
	SCA	2.87E−11	Yes	4.44E−16	20.195
	WOA	1.07E−06	Yes	4.44E−16	4.00E−15
	GJO	2.87E−11	Yes	4.44E−16	4.00E−15
F11	SHO	1	No	0	0
	GWO	0.5059	No	0	0
	SCA	2.87E−11	Yes	0	0.939169
	WOA	1	No	0	0
	GJO	1	No	0	0
F12	SHO	2.87E−11	Yes	0.014474	0.399372
	GWO	4.73E−11	Yes	0.014474	0.057836
	SCA	2.87E−11	Yes	0.014474	25.2745
	WOA	1.93E−05	Yes	0.014474	0.027111
	GJO	2.87E−11	Yes	0.014474	0.244984
F13	SHO	2.87E−11	Yes	0.301756	2.50339
	GWO	0.000978	Yes	0.301756	0.600356
	SCA	2.87E−11	Yes	0.301756	2,395.28
	WOA	1.21E−05	Yes	0.301756	0.624412
	GJO	4.84E−10	Yes	0.301756	1.70803
F14	SHO	1.62E−09	YES	0.998004	2.98211
	GWO	2.87E−11	YES	0.998004	12.6705
	SCA	2.87E−11	YES	0.998004	1.99976
	WOA	0.8941	Yes	0.998004	1.49502
	GJO	2.87E−11	Yes	0.998004	2.98211
F15	SHO	0.002322	Yes	0.00031	0.000331
	GWO	1.62E−09	Yes	0.00031	0.000677
	SCA	1.77E−09	Yes	0.00031	0.000887
	WOA	2.08E−06	Yes	0.00031	0.00041
	GJO	0.002322	Yes	0.00031	0.00041
F16	SHO	2.86E−05	Yes	−1.03163	−1.03163
	GWO	2.87E−11	Yes	−1.03163	−1.03163
	SCA	2.87E−11	Yes	−1.03163	−1.03157
	WOA	8.12E−09	No	−1.03163	−1.03163
	GJO	3.51E−11	Yes	−1.03163	−1.03163
F17	SHO	7.03E−11	Yes	0.397887	0.398343
	GWO	2.87E−11	Yes	0.397887	0.397889
	SCA	2.87E−11	Yes	0.397887	0.399506
	WOA	0.000145	No	0.397887	0.397887
	GJO	2.87E−11	Yes	0.397887	0.397903
F18	SHO	0.01801	Yes	3	3
	GWO	3.50E−08	Yes	3	3
	SCA	5.77E−11	Yes	3	3.00001
	WOA	8.12E−09	No	3	3
	GJO	5.39E−07	Yes	3	3
F19	SHO	2.87E−11	Yes	−3.86278	−3.85338
	GWO	2.87E−11	Yes	−3.86278	−3.85491
	SCA	2.87E−11	Yes	−3.86278	−3.85445
	WOA	8.12E−09	No	−3.86278	−3.86278
	GJO	7.03E−11	Yes	−3.86278	−3.8549
F20	SHO	2.87E−11	Yes	−3.32197	−2.99108
	GWO	7.43E−05	Yes	−3.32197	−3.11006
	SCA	1.15E−10	Yes	−3.32197	−3.01884
	WOA	0.7562	Yes	−3.32197	−3.2031
	GJO	5.31E−08	Yes	−3.32197	−3.10868
F21	SHO	2.87E−11	Yes	−10.1532	−4.53399
	GWO	3.18E−11	Yes	−10.1532	−10.1511
	SCA	2.87E−11	Yes	−10.1532	−1.85633
	WOA	0.04595	Yes	−10.1532	−5.0552
	GJO	2.87E−11	Yes	−10.1532	−10.1316
F22	SHO	2.87E−11	Yes	−10.4029	−4.6449
	GWO	3.51E−11	Yes	−10.4029	−10.4008
	SCA	2.87E−11	Yes	−10.4029	−2.84811
	WOA	0.004969	Yes	−10.4029	−5.08767
	GJO	2.87E−11	Yes	−10.4029	−10.3887
F23	SHO	2.87E−11	Yes	−10.5364	−4.64775
	GWO	7.76E−11	YES	−10.5364	−10.5345
	SCA	2.87E−11	YES	−10.5364	−3.80473
	WOA	0.000347	YES	−10.5364	−5.12848
	GJO	2.87E−11	YES	−10.5364	−10.5228

**TABLE 5:** Wilcoxon rank-sum test results comparing moSHO to other algorithms across 23 benchmark functions.

	Total comparisons	moSHO better (p < 0.05)	Percentage (%)
**GJO**	23	20	87.0
**GWO**	23	19	82.6
**SCA**	23	23	100.0
**SHO**	23	20	87.0
**WOA**	23	12	52.2

Moreover, the statistical findings prove the earlier observations based on mean and variance analysis. While algorithms such as GWO and WOA occasionally produced competitive results, their improvements did not reach statistical significance in most scenarios. Similarly, SCA and GJO failed to consistently challenge moSHO’s superiority, reflecting their weaker balance between exploration and exploitation. By contrast, the hybrid and multi-strategy mechanisms embedded in moSHO provided it with a decisive advantage, enabling both precise exploitation of unimodal functions and sustained exploration of multimodal and composite problems. Taken together, these results confirm that moSHO’s design not only enhances average performance but also ensures statistically validated robustness and reliability across the CEC benchmark suite.

### 4.4 moSHO analyses on benchmark functions

**FIGURE 1:**
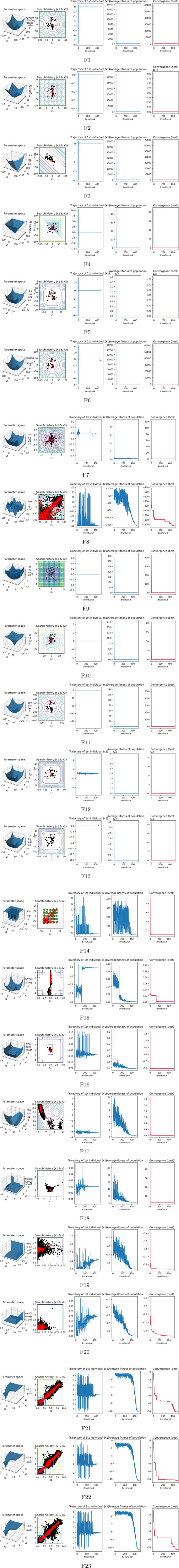
Behavior and movement analysis on CEC 2014.

The visualizations provided for functions F1–F23 offer a comprehensive depiction of the optimization dynamics of moSHO. Figure 1 illustrates the search space and convergence attributes across all functions in CEC 2014. The parameter space and search history plots reveal that the algorithm rapidly guides the population toward promising regions of the landscape while maintaining a controlled level of exploration. The trajectory of representative individuals illustrates the balance between diversification in the early stages and intensified exploitation as iterations progress. Moreover, the fitness evolution curves consistently exhibit a sharp decline in both average and best fitness values within the initial iterations, followed by stabilization at near-optimal solutions. These patterns confirm that moSHO converges efficiently while preserving robustness across unimodal, multimodal, and composite problem settings.

### 4.5 Ablation study

The ablation results reported in Table 6 demonstrate the contribution of each individual component within the proposed moSHO framework. Across unimodal (F1–F4), multimodal (F8, F10, and F12), composite (F13–F16), and hybrid composition functions (F20–F23), incorporating the FAD mechanism consistently improves the baseline SHO by enhancing population diversity and exploration. The integration of the best–worst mutation strategy further strengthens exploitation, leading to improved solution quality on more complex landscapes. In addition, the QRBL strategy contributes to maintaining search diversity by introducing quasi-opposite candidate solutions, which complement the exploration capability of FADs and support a smoother transition toward exploitation.

**TABLE 6:** Ablation study on selected CEC2014 benchmark functions with mean and standard deviation values over 30 independent runs.

	SHO		SHO + FADs		SHO + FADs + BWM		moSHO	
Function	Mean	Std	Mean	Std	Mean	Std	Mean	Std
F1	1.09e−171	–	1.72e−181	–	1.16e−180	–	**0.00**	–
F2	2.29e−92	1.22e−91	4.19e−98	1.99e−97	3.54e−97	1.68e−96	**0.00**	0.00
F3	7.04e−132	2.01e−131	1.36e−133	7.43e−133	4.43e−132	2.24e−131	**0.00**	0.00
F4	3.02e−75	1.10e−74	1.87e−77	1.00e−76	1.74e−78	7.14e−78	4.94e−324	–
F8	−7.09e+02	7.56e+01	−8.30e+02	8.89e+01	−1.00e+03	8.23e+01	−1.10e+03	1.45e+02
F10	2.10e−15	1.80e−15	2.46e−15	1.79e−15	2.10e−15	1.80e−15	4.44e−16	–
F12	4.08e−01	1.33e−01	1.58e−02	7.28e−03	1.25e−02	6.66e−03	1.57e−02	8.65e−03
F13	2.50	0.18	0.50	0.21	0.47	0.24	**0.41**	0.37
F14	5.49	4.72	1.30	0.65	0.998	–	**0.998**	–
F15	4.04e−04	2.59e−04	3.53e−04	2.09e−04	3.88e−04	2.91e−04	3.86e−04	2.75e−04
F16	−1.03	3.68e−09	−1.03	9.17e−11	−1.03	3.04e−11	−1.03	8.88e−10
F20	−2.87	0.28	−3.26	0.07	−3.26	0.07	−3.26	0.06
F21	−4.11	1.21	−6.07	2.07	−6.33	2.38	−10.2	9.14e−05
F22	−4.29	1.14	−6.46	2.43	−7.21	2.65	−10.4	1.56e−04
F23	−4.56	0.43	−6.37	2.34	−6.75	2.52	−10.5	1.58e−04

Overall, the full moSHO variant achieves the best mean performance on most of the tested functions, confirming the complementary and synergistic effect of the proposed strategies.

To further illustrate the effect of each component on the optimization dynamics, convergence curves of the ablation variants are presented for representative multimodal and composite benchmark functions in Figures 1–9. These functions are selected as they better highlight the differences in exploration and exploitation behaviors among the compared variants.

**FIGURE 2:**
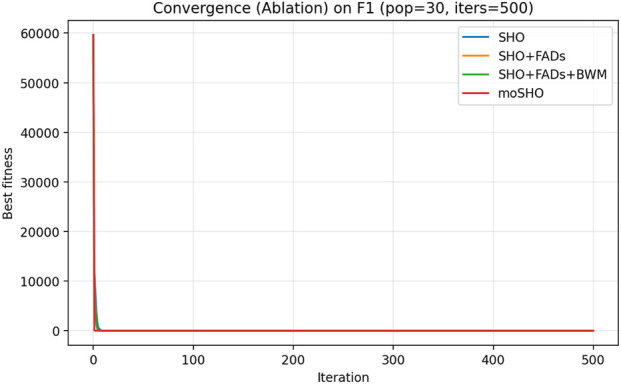
Convergence curves of the ablation variants on CEC2014 F1 (30 runs, best-so-far).

**FIGURE 3:**
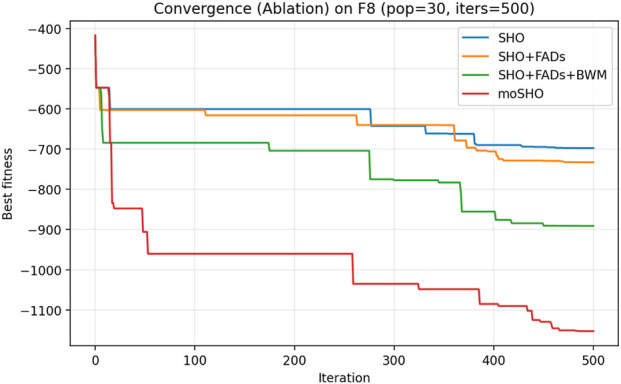
Convergence curves of the ablation variants on CEC2014 F8 (30 runs, best-so-far).

**FIGURE 4:**
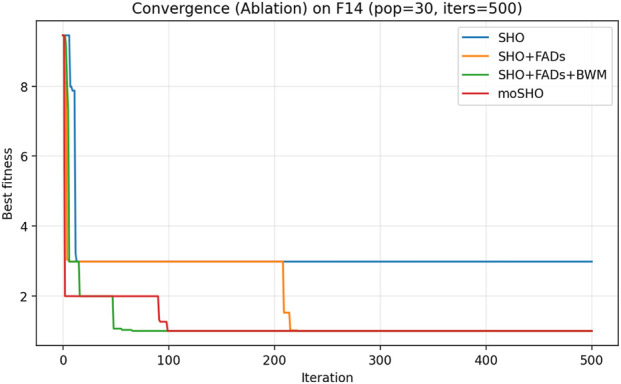
Convergence curves of the ablation variants on CEC2014 F14 (30 runs, best-so-far).

**FIGURE 5:**
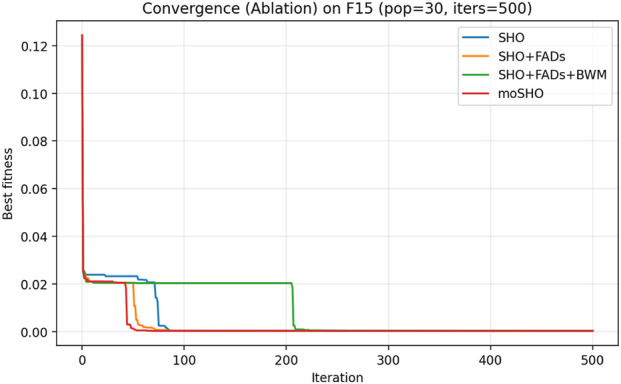
Convergence curves of the ablation variants on CEC2014 F15 (30 runs, best-so-far).

**FIGURE 6:**
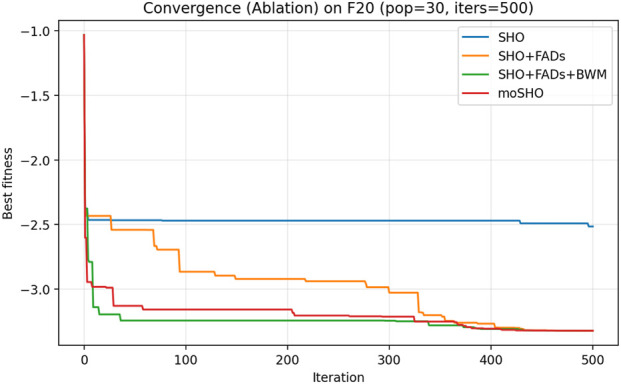
Convergence curves of the ablation variants on CEC2014 F20 (30 runs, best-so-far).

**FIGURE 7:**
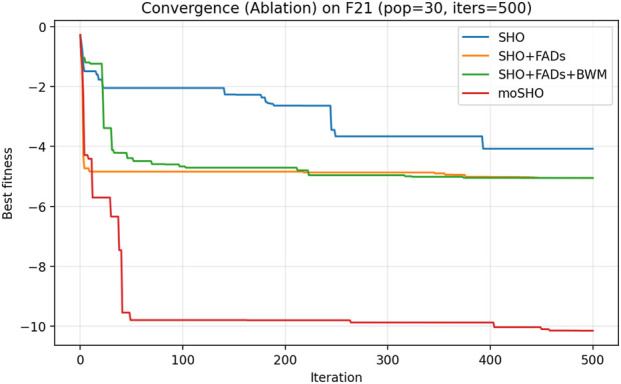
Convergence curves of the ablation variants on CEC2014 F21 (30 runs, best-so-far).

**FIGURE 8:**
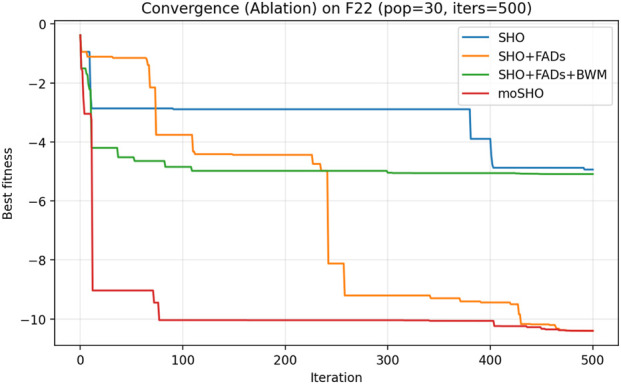
Convergence curves of the ablation variants on CEC2014 F22 (30 runs, best-so-far).

**FIGURE 9:**
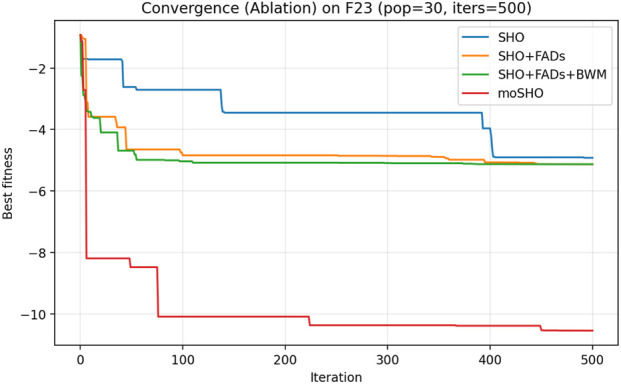
Convergence curves of the ablation variants on CEC2014 F23 (30 runs, best-so-far).

### 4.6 Convergence curve for CEC2014

**FIGURE 10:**
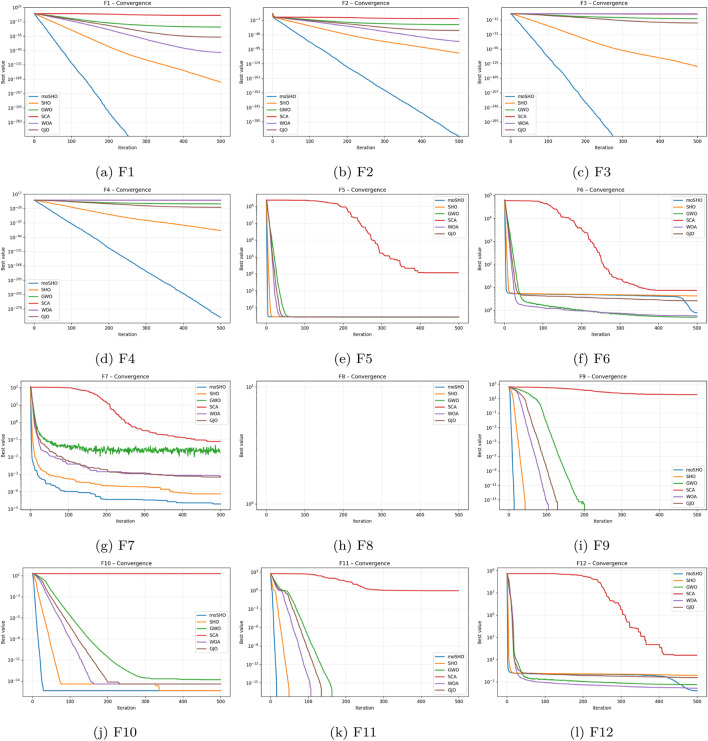
Convergence curves for the CEC2014 benchmark functions **(a–l)** F1–F12.

**FIGURE 11:**
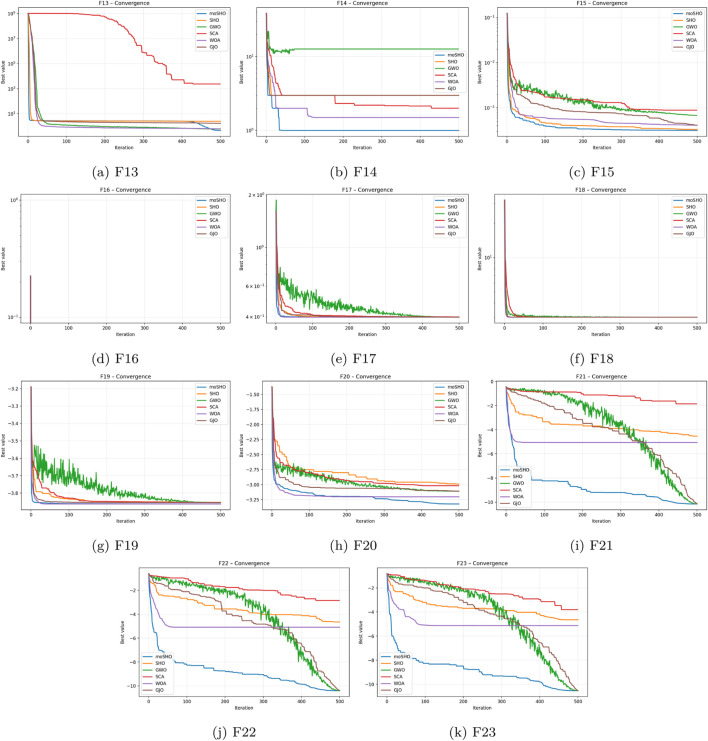
Convergence curves for the CEC2014 benchmark functions **(a–k)** F13–F23.

The convergence curves presented in Figures 10, 11 for the benchmark functions F1–F23 provide a clear illustration of the comparative optimization dynamics of the tested algorithms. In nearly all cases, moSHO exhibits a markedly faster reduction in objective function value, converging toward the global optimum significantly earlier than its competitors. The steep initial decline observed in moSHO’s trajectory demonstrates its strong exploitation capability, while its sustained improvement across iterations indicates an effective balance with exploration. By contrast, algorithms such as SHO, GWO, and WOA converge more slowly and plateau at higher fitness values, suggesting susceptibility to premature convergence. SCA consistently lags with minimal progress, highlighting its instability across diverse landscapes. Overall, the convergence patterns in Figures 10, 11 confirm that the hybrid and multi-strategy design of moSHO ensures both rapid and robust convergence across unimodal, multimodal, and composite functions.

### 4.7 Boxplots (F1–F23)

**FIGURE 12:**
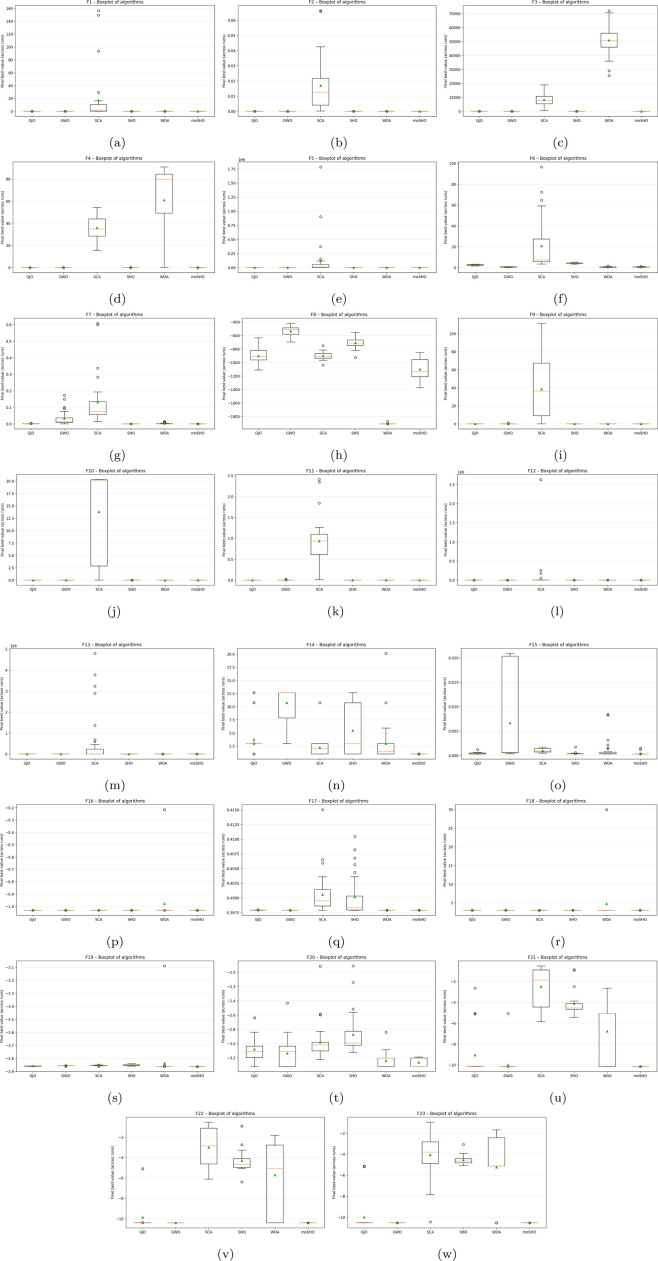
Boxplots of the final best values obtained by all algorithms for the CEC2014 benchmark functions **(a–w)** F1–F23.

The boxplots illustrated in Figure 12 provide further evidence of the comparative stability of the algorithms across multiple independent runs. As shown, moSHO consistently achieves the lowest distribution of final best values with minimal variance, confirming its robustness and repeatability. In contrast, SCA exhibits the widest spread with numerous outliers, reflecting its instability and tendency toward inconsistent convergence. The original SHO, along with GWO, WOA, and GJO, shows a relatively tighter distribution than SCA, yet the medians remain noticeably higher than that of moSHO, indicating inferior accuracy. Overall, the results in Figure 12 prove that moSHO has superior average performance and a high degree of reliability across stochastic runs.

### 4.8 Exploration–exploitation analysis

This subsection analyzes the balance between exploration and exploitation achieved by the proposed strategies. To this end, the performance of different components is evaluated through the ablation study based on the SHO algorithm. The results indicate that the FAD mechanism significantly enhances exploration in the early iterations by increasing population diversity and mitigating premature convergence, which is reflected by slower yet more robust initial convergence than the baseline SHO. In contrast, the best–worst mutation strategy strengthens exploitation in later stages, leading to faster convergence and improved solution quality. By effectively combining these two complementary behaviors, the full moSHO framework achieves sufficient global exploration in the early stages and strong exploitation as the search progresses. In addition, the QRBL strategy further enhances exploration by introducing quasi-opposite candidate solutions, which help the population escape local optima and improve search diversity, particularly during the intermediate stages of the optimization process.

As a result, moSHO consistently outperforms its ablation variants and baseline algorithms, particularly on multimodal and composite benchmark functions. Figure 1 depicts the convergence behavior CEC2014 F1 function.

Figure 3 illustrates the convergence behavior of the ablation variants on the multimodal CEC2014 F8 function. While the baseline SHO exhibits rapid early convergence, it tends to stagnate at suboptimal solutions, indicating insufficient exploration. Variants incorporating the FAD mechanism maintain stronger exploration in the early iterations, whereas the full moSHO achieves faster convergence in later stages due to enhanced exploitation. Similar trends are observed on composite benchmark functions, such as F14 (Figure 4), further demonstrating the effectiveness of the proposed exploration–exploitation balance. Figures 5–9 represents the convergence behavior of functions F15, F20, F21, F22, F23, respectively.

### 4.9 Time complexity analysis

Let N denote the population size, D the problem dimension, and T the maximum number of iterations. In each iteration, the baseline SHO performs position updates and boundary handling for all agents, which require O(ND) time, followed by fitness evaluations with a cost of $O\left(NC_{f}\right)$ , where $C_{f}$ denotes the cost of one objective evaluation.

The proposed moSHO introduces three additional strategies. The FAD mechanism applies adaptive, range-aware perturbations at the vector level, incurring O(ND) time per iteration. The BWPM operator performs Gaussian perturbations on the best individuals and differential updates with Cauchy perturbation for the worst individuals, which together also require O(ND) time. Finally, the QRBL strategy generates quasi-opposite candidates through vector operations, resulting in an additional O(ND) cost per iteration.

Because all three strategies operate at the vector level and do not increase the number of fitness evaluations, the overall time complexity of moSHO remains $O\;\left(T\cdot \left(ND+NC_{f}\right)\right)$ , which is the same asymptotic order as the baseline SHO, with only a modest constant-factor overhead.

## 5 UAV path planning case study

In this case study, the performance of the proposed moSHO is evaluated on a three-dimensional unmanned aerial vehicle (UAV) path planning task within an environment containing multiple static obstacles (Kumar et al., 2025). The UAV is required to navigate from a starting point to a goal destination while ensuring safe and efficient movement through the search space.

The environment is modeled as a bounded three-dimensional workspace in which obstacles are represented by simple geometric primitives such as cylinders or spheres. These obstacles are positioned in various configurations to create different levels of navigational difficulty. The UAV must plan a collision-free path that maintains a safe distance from all obstacles, minimizes travel distance, and avoids unnecessary maneuvers.

A two-threshold collision model is considered to represent realistic operational challenges: one threshold establishes the physical boundary of an obstacle, serving as a hard constraint, while the other adds a safety margin that penalizes paths that pass too close to obstacles (Cheng et al., 2025). This promotes solutions that provide safer navigation margins and prevent collisions.

The scenarios tested vary in obstacle density, spatial distribution, and start–goal positioning. Some environments present relatively open spaces with few obstacles, whereas others require the UAV to perform complex maneuvers through narrow passages. These different scenarios ensure a comprehensive evaluation of the algorithms’ ability to handle various path planning challenges.

### 5.1 Problem statement

A constrained three-dimensional optimization problem is used to formulate the UAV path planning task (Kumar et al., 2025). The goal is to determine the best waypoint sequence that connects the start position S to the goal position G while meeting safety and constraint requirements in a bounded workspace with predefined static obstacles.

Scenarios

Several path planning scenarios, each with a different configuration of obstacles and start–goal positions, are considered to evaluate the proposed approach. The movement difficulty, obstacle density, and spatial distribution vary between the scenarios. The environment settings are summarized in Table 7.

**TABLE 7:** UAV path planning scenarios.

Scenario	Start position	Goal position	Number of obstacles
1	(2.0, 2.0, 1.5)	(28.0, 28.0, 2.0)	4
2	(2.0, 2.0, 1.5)	(28.0, 28.0, 2.0)	6
3	(2.0, 2.0, 1.5)	(28.0, 28.0, 2.0)	8

**TABLE 8:** Obstacle parameters for each UAV path planning scenario.

Scenario	Obstacle ID	Center (x,y) [m]	Radius (m)
1	1	(11.0, 10.0)	3.0
1	2	(18.0, 6.0)	2.5
1	3	(15.0, 22.0)	3.0
1	4	(24.0, 22.0)	2.2
2	1	(11.0, 10.0)	3.0
2	2	(18.0, 6.0)	2.5
2	3	(15.0, 22.0)	3.0
2	4	(24.0, 22.0)	2.2
2	5	(11.0, 25.0)	2.8
2	6	(26.0, 8.0)	2.5
3	1	(11.0, 10.0)	3.0
3	2	(18.0, 6.0)	2.5
3	3	(15.0, 22.0)	3.0
3	4	(24.0, 22.0)	2.2
3	5	(11.0, 25.0)	2.8
3	6	(26.0, 8.0)	2.5
3	7	(11.0, 13.0)	3.0
3	8	(16.0, 7.0)	3.0

Obstacle avoidance

Obstacles are modeled as cylinders extending vertically through the flight volume, making vertical bypass impossible. This mechanism ensures collision-free navigation and improved clearance for safer flight. Obstacle parameters for each scenario is given Table 8 a two-threshold collision model is applied:

- Hard constraint: Direct collision with an obstacle is strictly forbidden. A path segment intersecting the obstacles’ physical radii is considered infeasible.
- Soft constraint: A safety margin is added around each obstacle. Paths entering this zone incur a penalty proportional to the squared violation distance.

Path representation

A candidate path P is defined as an ordered set of $n_{\text{wp}}$ waypoints:

$$P=\left\{p_{1},p_{2},\ldots ,p_{n_{\text{wp}}}\right\},\;p_{i}=\left(x_{i},y_{i},z_{i}\right),$$

where the first and last waypoints correspond to S and G , respectively, and the intermediate waypoints are decision variables optimized by the metaheuristic algorithm. The path is evaluated by connecting waypoints sequentially using straight-line segments.

Objective function

The total cost F(P) of a path is expressed as

$$F\;\left(P\right)=w_{\ell }\cdot L\;\left(P\right)+w_{\text{obs}}\cdot C_{\text{obs}}\left(P\right)+w_{\text{smo}}\cdot S\;\left(P\right)+w_{\text{alt}}\cdot C_{\text{alt}}\left(P\right),$$

where:

-
L(P) : total Euclidean path length,
-
$C_{\text{obs}}\left(P\right)$ : obstacle avoidance penalty (hard and soft),
-
S(P) : smoothness penalty based on turning angles,
-
$C_{\text{alt}}\left(P\right)$ : altitude violation penalty.
-
$w_{\ell }$ , $w_{\text{obs}}$ , $w_{\text{smo}}$ , and $w_{\text{alt}}$ are weights of the objective function.

#### 5.1.1 Weight sensitivity analysis

The weighting coefficients of the objective function were selected to balance path length, obstacle avoidance, smoothness, and altitude constraints. To verify robustness, additional experiments with perturbed weight settings were conducted, and consistent performance trends were observed. These results indicate that the proposed moSHO is not overly sensitive to moderate weight variations and maintains stable path planning behavior across different objective configurations.

#### 5.1.2 Path smoothness and practical considerations

The proposed moSHO generates waypoint-based trajectories by optimizing intermediate control points subject to obstacle avoidance constraints. Accordingly, the optimization process prioritizes feasibility, safety, and path optimality rather than explicit geometric smoothness, and the reported trajectories correspond to raw waypoint solutions. In practical UAV systems, path smoothness is commonly handled as a post-processing step, where techniques such as spline interpolation can be applied to the optimized waypoints to obtain dynamically feasible and smooth trajectories without affecting collision avoidance. This separation allows a fair evaluation of the optimization performance of moSHO while remaining consistent with real-world UAV navigation pipelines.

Key optimization factors

To achieve the minimum-cost path, the optimization process must balance:

1. **Path length:** shorter paths are preferred for efficiency.
2. **Safety margin:** avoiding proximity to obstacles reduces collision risk.
3. **Smoothness:** reducing sharp turns improves energy efficiency and stability.
4. **Feasibility:** paths must remain within the workspace boundaries.

These factors collectively define a trade-off surface, where the optimal solution depends on the scenario-specific environment and obstacle layout.

Algorithms and parameters

Six metaheuristic algorithms were tested: moSHO, SHO, GWO, SCA, WOA, and GJO. All algorithms share the same dimensionality of 30 decision variables (ten waypoints, each with (x,y,z) coordinates). The common parameters were:

- Population size: 30
- Maximum iterations: 200
- Random seed: fixed for reproducibility
- Bounds: $\left[x_{\min},x_{\max}\right]=\left[0,30\right]$ , $\left[y_{\min},y_{\max}\right]=\left[0,30\right]$ , $\left[z_{\min},z_{\max}\right]=\left[0,15\right]$

The weights of the objective function were set to $w_{\ell }=1.0$ , $w_{\text{obs}}=50.0$ , $w_{\text{smo}}=0.5$ , and $w_{\text{alt}}=10.0$ for all algorithms.

For each scenario, the path obtained by the best-performing algorithm is illustrated in a mixed-scene view (combined 3D, XY, and XZ projections). The other algorithms are represented with their best XY-projection path. Additionally, an overlay plot compares the trajectories of all algorithms, and convergence curves depict their optimization behavior.

### 5.2 Results and discussion

This section presents the results of the UAV path planning experiments for the three scenarios defined in Table 7. Six metaheuristic algorithms are compared: moSHO, SHO, GWO, SCA, WOA, and GJO. For each scenario, we report the best cost value, collision-free status, and convergence behavior.

#### 5.2.1 Scenario 1

**FIGURE 13:**
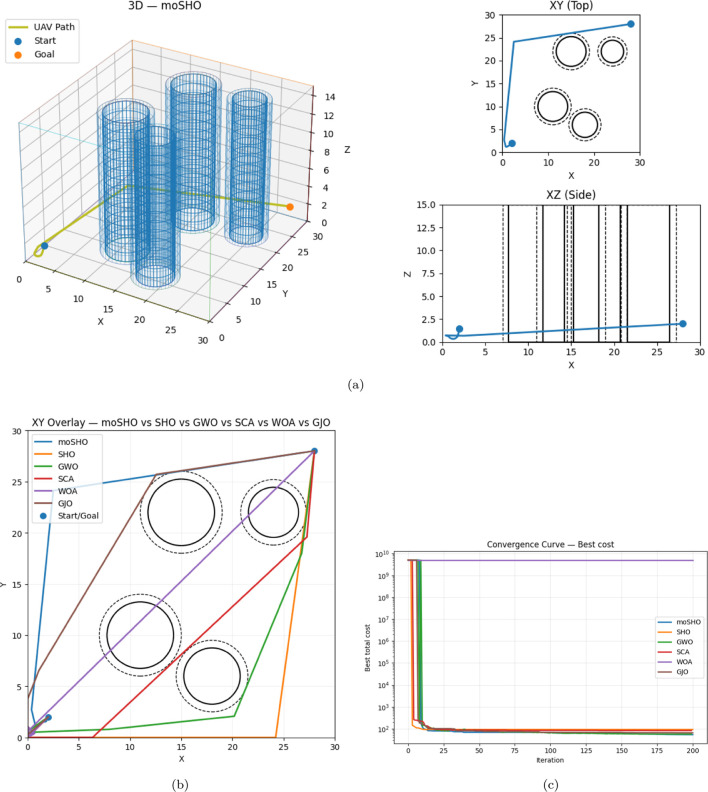
Scenario 1: Best path (top), overlay, and convergence (bottom). **(a)** Best trajectory (moSHO): 3D + XY + XZ. **(b)** XY overlay: all algorithms. **(c)** Convergence (log scale).

Table 9 shows that moSHO achieved the lowest cost (53.6934) with a collision-free path. GWO ranked second (56.3473), also collision-free. WOA failed to avoid collisions and received the maximum penalty. The smallest clearance observed was 0.7255 m (segment 10 near obstacle 2). Figure 13 shows the effectiveness of moSHO in Scenario 1 through the best trajectory, comparative overlays, and convergence performance.

**TABLE 9:** Scenario 1 results (best cost values and collision-free status).

Algorithm	Best cost	Collision-free?
moSHO	53.6934	Yes
SHO	92.9566	Yes
GWO	56.3473	Yes
SCA	82.3230	Yes
WOA	$5.0\times 10^{9}$	No
GJO	65.0709	Yes

#### 5.2.2 Scenario 2

**FIGURE 14:**
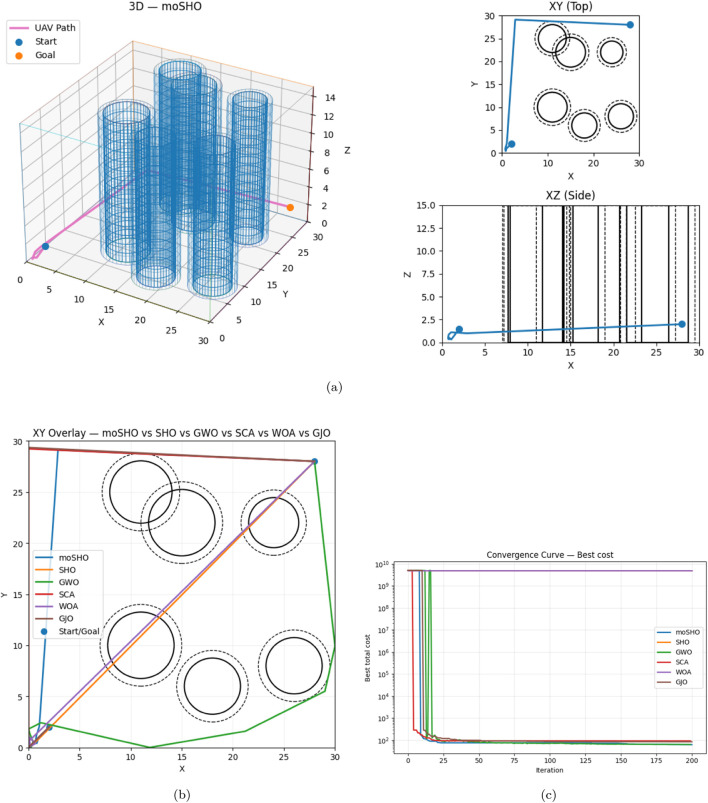
Scenario 2: **(a)** Best trajectory (moSHO): 3D + XY + XZ views. **(b)** XY overlay: all algorithms. **(b)** Convergence (log scale).

Table 10 shows moSHO again leading with a cost of 62.0605 (collision-free). GWO (63.1064) followed closely, also collision-free. SHO and WOA collided with obstacles and were penalized. The worst clearance was 0.7018 m (segment 10, obstacle 4). Figure 14 highlights the performance of moSHO in Scenario 2 through the optimal trajectory, comparative path overlays, and convergence characteristics.

**TABLE 10:** Scenario 2 results (best cost values and collision-free status).

Algorithm	Best cost	Collision-free?
moSHO	62.0605	Yes
SHO	$5.0\times 10^{9}$	No
GWO	63.1064	Yes
SCA	91.7067	Yes
WOA	$5.0\times 10^{9}$	No
GJO	83.6890	Yes

**TABLE 11:** Scenario 3 results (best cost values and collision-free status).

Algorithm	Best cost	Collision-free?
moSHO	60.3110	Yes
SHO	$5.0\times 10^{9}$	No
GWO	61.8416	Yes
SCA	91.7067	Yes
WOA	$5.0\times 10^{9}$	No
GJO	73.6150	Yes

#### 5.2.3 Scenario 3

**FIGURE 15:**
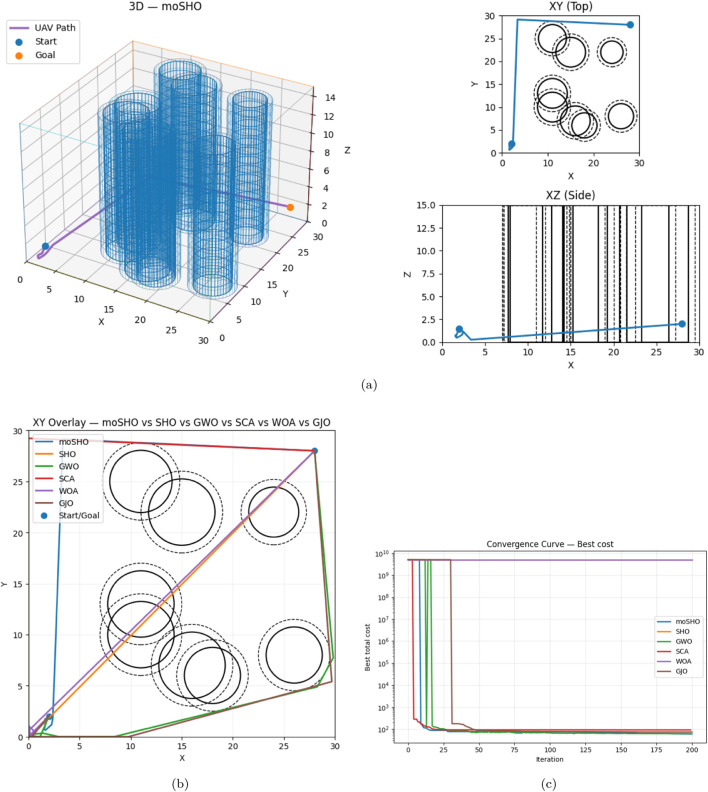
Scenario 3: Best path (top), overlay, and convergence (bottom). **(a)** Best trajectory (moSHO): 3D + XY + XZ. **(b)** XY overlay: all algorithms. **(c)** Convergence (log scale).

In the densest environment, Table 11 shows **moSHO** obtained results on the scenario 3 achieved a cost of 60.3110 (collision-free), outperforming GWO (61.8416). SHO and WOA collided with obstacles. The worst clearance was 0.7306 m (segment 10, obstacle 4). Figure 15 demonstrates the robustness of moSHO in Scenario 3 through the best trajectory, comparative overlays, and convergence behavior.

Across all scenarios:

- moSHO consistently produced the best solutions with full collision avoidance.
- GWO was competitive but slightly less effective in dense environments.
- SHO, SCA, and GJO were less consistent, often yielding higher costs.
- WOA frequently failed to generate feasible paths.
- The two-threshold collision model effectively penalized unsafe paths while allowing near-miss flexibility.

These results confirm moSHO’s strong potential for real-world UAV path planning.

## 6 Conclusion and future work

In this study, we proposed an enhanced variant of the Sea Horse Optimizer (SHO), termed moSHO, which integrates multiple strategies to improve exploration–exploitation balance and prevent premature convergence. Extensive experiments on the CEC2014 benchmark functions demonstrated that moSHO consistently outperforms classical and state-of-the-art algorithms. It achieved the lowest mean across all functions and provided statistically significant improvements in more than 80% of pairwise Wilcoxon rank–sum tests. The convergence and boxplot analyses further confirmed that moSHO yields superior accuracy and exhibits robustness and stability across multiple independent runs.

The effectiveness of moSHO was further validated in the context of UAV path planning, a challenging real-world application characterized by high-dimensional and constrained optimization requirements. Experimental results showed that moSHO can generate collision-free, smooth, and energy-efficient trajectories while adapting to dynamic environments and obstacle distributions. Compared to existing metaheuristics, moSHO demonstrated faster convergence toward optimal paths and stronger consistency across multiple trials, confirming its suitability for autonomous navigation tasks.

Even though the proposed method performs well, several directions for further study remain. Examining hybridization with reinforcement learning techniques, such as deep Q-networks, to improve adaptive decision-making during path planning is one exciting avenue. Deploying moSHO in multi-UAV cooperative missions, where swarm coordination and communication limitations add complexity, could be another extension. The algorithm’s relevance to actual UAV operations would be enhanced by adding real-time uncertainty modeling, such as dynamic wind conditions or communication delays. In conclusion, moSHO offers a reliable and effective solution that improves metaheuristic optimization performance and tackles real-world issues in UAV navigation. To close the gap between theoretical optimization and actual autonomous aerial systems, future efforts will focus on improving adaptability, scalability, and integration with intelligent control systems.

## Data Availability

The original contributions presented in the study are included in the article/Supplementary Material; further inquiries can be directed to the corresponding author.
